# Adjuvant chemotherapy for node negative, high Recurrence Score^TM^ breast cancer: in defense of de-escalation

**DOI:** 10.1038/s41523-019-0119-5

**Published:** 2019-08-12

**Authors:** Diana Lake, Andrew D. Seidman

**Affiliations:** Breast Medicine Service, Department of Medicine, Memorial Sloan Kettering Cancer Center, New York, NY USA

**Keywords:** Breast cancer, Breast cancer

Recent clinical trials have demonstrated the ability to de-escalate therapy for early stage breast cancer regardless of whether the intervention is surgical,^1–3^ radiotherapeutic,^4^ or systemic.^5–8^ The expectation of improved outcomes with contemporary therapy coupled with a desire to minimize toxicity have motivated these efforts. Patients with early breast cancer no longer only wish to be cured and have their breast conserved but also avoid lymphedema, unnecessary radiotherapy, and many toxicities that have historically accompanied cytotoxic chemotherapy. Witness the rising use of scalp cooling devices to mitigate against alopecia,^9^ the use of icing of hands and feet to avoid nail toxicity,^10–12^ and strategies to minimize taxane-associated peripheral neuropathy.^13–15^

The initial demonstration that patients with HR+/HER2−, node negative high 21-gene Recurrence Score^TM^ (RS) breast cancer benefit from adjuvant chemotherapy showed a 28% absolute reduction in distant recurrence when either cyclophosphamide, methotrexate, and fluorouracil (CMF) or MF were administered as an addition to tamoxifen.^16^ In the TAILORx trial,^17^ where medical oncologists were given the opportunity to choose the chemotherapy regimen for patients with T1-2N0, HR+/HER2− breast cancer, the selections for those with RS >25 were: docetaxel/cyclophosphamide (TC) 45%, anthracycline ± taxane: 26%, anthracycline + taxane: 19%, other/not specified: 6%, and CMF: 4%. For patients with RS <25 randomized to chemotherapy (which did not improve outcomes), only 8% chose CMF. This begs a specific question: is the incremental toxicity associated with anthracycline and/or taxane administration in this population of patients with HR+/HER2−, node negative breast cancer, as compared to CMF, warranted?

Logically, answering this would require a direct randomized trial of an anthracycline and/or taxane-containing regimen versus CMF in this population. Such a trial does not exist and is not likely to be conducted. The alternative approach is to identify randomized trials that address that question and use risk models to extrapolate to the specific population in question. Again, such studies exist for anthracycline regimens versus CMF but not for taxanes. Finally, and least satisfying, one must make indirect and cross-study comparisons to attempt to estimate relative benefits. (It is fair to stipulate the differences in toxicity since those are not histology or anatomy dependent, unlike benefits).

Now, consider the patient with RS of 28 with an average-sized tumor and negative nodes. The estimated absolute reduction in 10-year risk of distant recurrence for CMF/MF over tamoxifen alone (5 years) is 5%. One might argue that anthracycline-based regimens would offer an incremental benefit over CMF, given the 4% reduction in recurrence and mortality observed across randomized trials of anthracycline versus CMF in 9527 patients the EBCTG meta-analysis.^18^ Notably, these patients had unknown HER2 status when randomized and largely received non-dose-dense anthracycline schedules. It is possible that the specific population with a high RS derives no such benefit since they are all HER2 negative (by definition) and a subsequent analysis of these data for 3818 patients with known HER2-negative disease after testing of archived tissue showed no benefit in disease-free or overall survival for anthracycline-based adjuvant therapy over CMF.^19^

One trial (USORT 9735) has demonstrated that four cycles of TC is superior to four cycles of AC, both administered in q-3 weekly cycles.^20^ Of note, more than half of the patients enrolled had node-positive disease, and more than a quarter had HR-negative breast cancer. Curiously, in a trial (ECOG 2197) 3-fold larger than this study, the combination of doxorubicin and docetaxel (AT) was not superior to the identical AC regimen. Furthermore, the DFS favored AT over AC for ER- patients (HR 1.24, 95% CI: 0.95–1.62), and AC for ER+ patients (HR 0.79, 95% CI: 0.60–1.03).^21^ No trial has compared TC to CMF, yet randomized trials involving over 5,000 patients comparing AC × 4 q 3 weeks to CMF showed no advantage for the anthracycline-based regimen.^18^ Finally, in the “ABC” trials (USOR 06–090, NSABP B-46-I/USOR 07132, and NSABP B-49/NRG Oncology), patients with node negative, ER+ breast cancer had a HR for DFS that trended strongly in favor of TC (×6), rather than AC plus taxane (0.69; 95% CI 0.39–1.19), with all other groups favoring AC plus taxane.^22^ It is hard to draw a conclusion that “more than CMF” or “more than AC” is advantageous since none of these trials provide a top line result confirming a clear and convincing benefit.

Might a taxane-containing regimen add enough benefit to AC × 4 in HR+/HER2− breast cancer patients to justify the sequential combination in patients with node negative, high RS disease? Among 1065 node-*positive*, HR+ patients with known RS enrolled in NSABP B-28, the likelihood ratio test for interaction between the use of paclitaxel or not did not show differential treatment effect across RS groups, arguing against escalation of chemotherapy as a function of RS, or a unique RS-paclitaxel benefit relationship.^23^ This argues that escalation of chemotherapy based on a specific RS value above 25 (e.g., 30 vs. 35 vs. 40) is not evidence-based.

So why was TC the most popular regimen employed in TAILORx, despite the differential toxicity expected as compared to CMF? For some patients, the issues of alopecia and neuropathy may be as fundamental as whether an assay directs the use of any chemotherapy versus none at all.^17,24–26^ Why are medical oncologists convinced that escalation from the regimen that originally demonstrated benefit in NSABP B-20 (CMF/MF) is warranted, in an era of de-escalation? Why don’t reports and lawsuits involving permanent alopecia with docetaxel, (https://drugsafetynews.com/2018/07/03/taxotere-permanent-hair-loss-warnings-were-issued-in-the-e-u-ten-years-earlier-that-in-the-u-s-why/) and hyper-lacrimation^27^ dampen the enthusiasm for TC in this clinical setting? One can only speculate, and these authors daresay that aggressive marketing of newer regimens and amnesia for older ones has some influence, along with the potential convenience of limiting treatment to just four cycles. In the recent West German Study Group PlanB trial, patients with N0/1 HR+/HER2— early breast cancer with RS > 11 were randomized to receive anthracycline + taxane versus taxane-based adjuvant chemotherapy (in addition to standard endocrine therapy)—CMF was not even an option.^28^

Finally, if one is to administer CMF—which specific regimen? The regimen demonstrating improved outcomes over tamoxifen alone in NSABP B-20 employed cyclophosphamide 100 mg/m^2^ orally daily on days 1–14 inclusive every 28 days for 6 cycles.^16,29^ Methotrexate 40 mg/m^2^ and 5-fluorouracil 600 mg/m^2^ were administered intravenously on days 1 and 8 every 28 days for 6 cycles. Patients receiving MF without C received M 100 mg/m^2^ and F 600 mg/m^2^ followed by leucovorin 15 mg/m^2^ orally every 6 h for six consecutive doses beginning 24 h after the administration of M. While large definitive randomized controlled trials (RCTs) of CMF dose and schedule in the adjuvant setting do not exist and are unlikely to ever be performed, retrospective analysis of dose intensity (DI) of adjuvant CMF regimens in breast cancer RCTs demonstrated that DI was an independent significant correlate of relapse-free survival in multivariate analysis.^29,30^ This is now substantiated in the more recent EBCTG patient-level meta-analysis of 37,298 women with early breast cancer in 26 randomized trials, where the impact of dose-density was further demonstrated.^31^ The feasibility and safety adjuvant dose-dense parenteral CMF with filgrastim has been well-described.^32^

CMF is not without toxicity. Myelosuppression, nausea, and mucosal toxicity not uncommon, but typically mild. In a large review of second malignancies following CMF-based adjuvant chemotherapy, including regimens that extended to 12 months, there was no evidence of a significantly increased risk of secondary malignancies, while the cumulative risk of acute non-lymphocytic leukemia was 0.23 ± 0.15%.^33^ The risk is associated with cumulative cyclophosphamide (C) dose, with all-parenteral regimens delivering a significantly lower C dose. Secondary myelodysplastic syndrome (MDS) and acute myeloid leukemia (AML) are more common with anthracycline-containing regimens than with CMF;^34,35^ regarding non-anthracycline regimens, the 95% confidence interval for incidence rate of secondary AML/MDS for TC and CMF in patients ≥65 years of age are overlapping, with longer follow-up needed for TC.^36^

In the absence of a direct comparison between chemotherapy regimens that represent escalation beyond the original NSABP B-20 validation study showing the benefit of CMF/MF over tamoxifen for N0, high RS early breast cancer, the interconnected evidence above (*vide supra*) and the dictum “primum non-nocere” argues strongly that there is plenty of room for chemotherapy de-escalation in this clinical setting, or no evidence for escalation in the first place. This is particularly relevant for older patients and those with comorbidities for whom the therapeutic index for anthracycline and/or taxane exposure may be narrower.
